# Treatment and Management of Shewanella algae Necrotizing Cellulitis

**DOI:** 10.7759/cureus.73680

**Published:** 2024-11-14

**Authors:** Alan Wang, Matthew Hibdon, Chakradhar Desaraju, Gustine Liu-Young

**Affiliations:** 1 Osteopathic Medicine, Nova Southeastern University Dr. Kiran C. Patel College of Osteopathic Medicine, Clearwater, USA; 2 Family Medicine, Tampa General Hospital, Crystal River, USA; 3 Infectious Disease, Tampa General Hospital, Crystal River, USA; 4 Infectious Disease, Hospital Corporation of America (HCA) Florida Citrus Hospital, Inverness, USA

**Keywords:** bacteremia, necrotizing cellulitis, shewanella algae, soft tissue infections, treatment

## Abstract

*Shewanella algae* is considered an emerging novel pathogenic cause of severe skin infections. The organism is a gram-negative motile bacillus commonly found in marine environments. While the more common causes of skin and soft tissue infections include *Staphylococcus aureus*, beta-hemolytic Streptococci, and/or *Vibrio vulnificus,* *S. algae* have also been isolated in rare cases. Notably, this organism is more likely to cause infection in patients with open wounds and is commonly seen in patients with underlying diabetes and peripheral vascular disease. In this case, a 71-year-old male patient who developed severe necrotizing cellulitis infection of the left lower limb complicated by bacteremia. The patient was immediately started on empiric treatment with intravenous antibiotics until *S. algae* was identified; thereafter, the antibiotic treatment was adjusted accordingly. During the course of hospitalization, the patient required serial surgical debridement procedures to achieve source control. The purpose of the case report is to increase awareness among healthcare professionals on how *S. algae* infections are contracted, the clinical presentation and effects this microbe has on patients with chronic comorbidities, along with the antibiotic treatment, as no guidelines have been established thus far.

## Introduction

*Shewanella algae* is a gram-negative, facultative anaerobic, oxidase-positive, rod-shaped, and motile organism commonly found in aquatic environments worldwide [1]. Infections caused by *S. algae*, however, are primarily reported in the warmer regions [2]. This emerging human pathogen can cause a wide range of infections, which often involve the skin and soft tissue, endocardium, and gastrointestinal tract. Additionally, this pathogen can lead to bacteremia and even septic shock [3]. The populations with the highest risk for *S. algae* infections are the elderly, immunocompromised individuals, and those with chronic comorbidities such as diabetes mellitus and peripheral arterial disease [2]. The case presented in this article occurred in the coastal region of Crystal River, Florida.

## Case presentation

**Figure 1:**
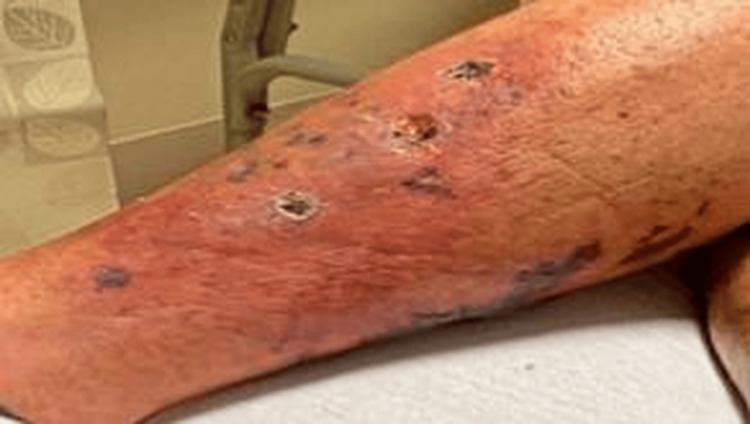
Initial assessment of the lesions on the left lower extremity

**Figure 2:**
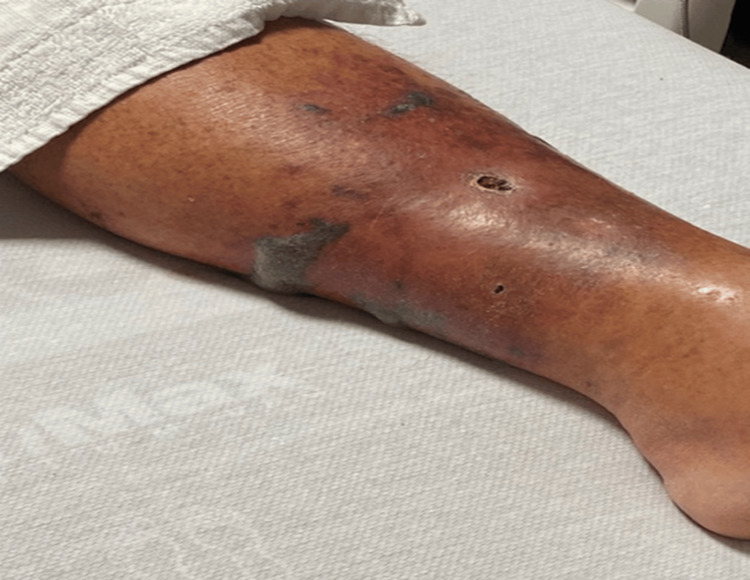
A fluid collection (bullae) was noted on the posteromedial left lower extremity during the second hospitalization day

**Figure 3:**
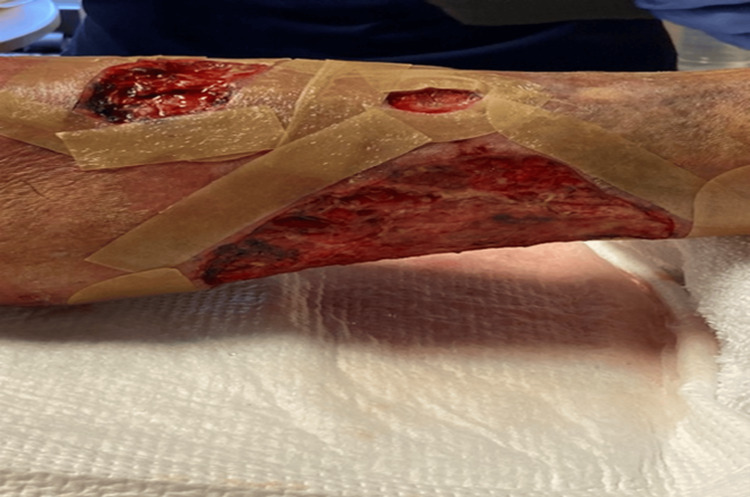
Ulcers on the left lower extremity after intravenous antibiotic treatment and the first surgical debridement

**Figure 4:**
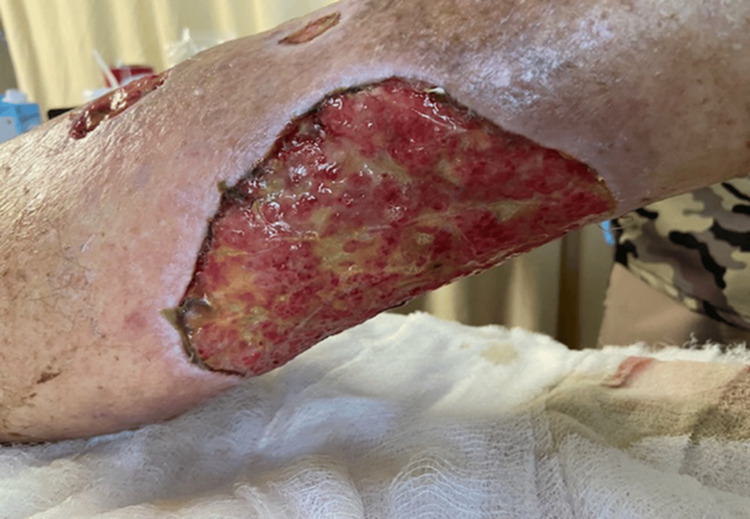
Ulcers on the left lower extremities after intravenous antibiotic treatment, second surgical debridement, and wound vacuum

A 71-year-old male patient with a past medical history of type II diabetes mellitus, peripheral vascular disease, hypertension, hyperlipidemia, chronic obstructive pulmonary disease, chronic atrial fibrillation, and deep vein thrombosis presented with left lower leg abrasions after falling off his bicycle two months before admission. He did not seek medical attention after this initial injury. The patient also stated that he spent some time in the waist-high water off the West Florida coast two weeks before falling. He could not remember if he sustained any leg injuries at that time. Over the course of two months, he applied topical antibiotic ointment on the abrasions; however, the wound did not improve. One day before visiting the emergency room, he noticed dark skin lesions scattered over the left lower calf circumferentially. In addition to these new lesions, he also had difficulty bearing weight and walking due to intolerable pain, which prompted him to seek medical treatment. On admission, the patient was afebrile. Blood pressure was 137/95 mm Hg, pulse was 92/minute, and respirations were 20/minute. Labs revealed a peripheral WBC count of 22,660/mm^3^ with neutrophilia and increased immature granulocytes, creatine phosphokinase at 91 mcg/L, and blood sugar at 193 mg/dL. Blood culture and wound culture obtained at admission revealed an atypical organism, but the microbiology laboratory did not perform routine sensitivity testing as there were no recommended sensitivity panels for it. Physical examination revealed circumferential left lower leg scattered dark superficial ulcerations suggestive of necrotizing infection. Palpation of the leg for crepitus was difficult due to severe pain in the area. Because of his clinical presentation of necrotizing soft tissue infection, he was started on IV piperacillin-tazobactam (Zosyn) and vancomycin, and later, medications were switched to clindamycin and ceftriaxone. An X-ray was done to rule out necrotizing fasciitis, and general surgery was consulted for debridement. The X-ray showed absence of gas formation, thus necrotizing fasciitis was ruled out. A CT scan showed left lower soft tissue edema and lateral skin irregularity. Antibiotic coverage was switched to clindamycin and ceftriaxone. Tissue culture results and sensitivities were delayed due to the innate characteristics of the organism. Over the course of the hospitalization, the patient's left leg became more edematous and inflamed up to the extent that a below-knee amputation was being considered if the infection progressed further. Once *S. algae* was identified by the lab, antibiotics were switched to IV ceftazidime and doxycycline for 14 days. The patient was closely monitored and required three wound debridement sessions throughout the hospital stay. In addition, a wound vacuum was applied. He did not require leg amputation with aggressive debridement and antibiotic treatment. The visual progression of the patient's left lower extremity is displayed in Figures 1-4.

## Discussion

Skin and soft tissue infections are routinely seen in hospital emergency rooms worldwide, and medical professionals are well aware of the various microbes present in these infections. Common commensal organisms such as *Staphylococcus aureus* and *Pseudomonas aeruginosa* can be easily identified and treated by medical professionals. However, treatment is far more complex and challenging with atypical infections, such as the one seen with *S. algae*.

The pathogenesis of *S. algae* is unknown; however, some investigators attribute its virulence to the production of alpha-hemolysins. This toxin alters the membrane permeability and causes cell lysis in a variety of human hosts [4]. Conveniently, *S. aureus* also produces alpha-hemolysins (also known as "alpha toxins"), which function by forming pores in cell membranes and aiding the pathogenesis of severe skin infections [5]. Other contributing factors thought to aid in the virulence of *S. algae* include siderophores, biofilm production, and iron-chelating compounds [6].

Our patient presented with a necrotizing soft tissue infection, which was characterized by scattered dark skin lesions in the left lower extremity. This infection was complicated by necrotic tissue and bullae formation (see Figure 2). Additionally, he has the classic risk factors and environmental exposure typical for the other reported *S. algae* infections. These risk factors are his chronic type II diabetes mellitus and peripheral vascular disease. As for environmental exposure, most *S. algae* cases have reported some form of saltwater exposure (this being the water itself or exposure to a saltwater fish) leading to this complicated infection. What makes our case different from the others are the treatment options used and the initial presentation of the wound. To date, no case of *S. algae* infections in Citrus County, Florida, can be searched on PubMed and Cureus, and it would become the third case reported on the west coast of Florida. The other two cases were reported in Tampa, Florida (2018), and Brooksville, Florida (2019) [7].

The patient was initially given piperacillin-tazobactam (Zosyn) and vancomycin. Due to rapid progression and bullae formation, antibiotics were tailored to suspected organisms, and General Surgery was consulted for debridement and tissue culture. Doxycycline was added to cover for atypical infection. Due to the slow growth of this atypical fastidious organism and the lack of availability of ceftazidime IV, antibiotic treatment was challenging. He was given gentamicin and levaquin temporarily. On the fifth day of hospitalization, the leg appeared to heal well and only showed some signs of lymphangitis. However, on the seventh day of hospitalization, new areas of concern developed despite appropriate antibiotic treatment. The patient required serial debridement sessions, and when IV ceftazidime became available, the patient was switched to IV ceftazidime and per ora doxycycline for 14 days. The patient continued to slowly improve and did not require any leg amputation.

While reviewing other case reports, all of which involved patients with similar skin and soft tissue infections, a trend became clear: there are no standardized treatment options for *S. algae*. This is because infections involving *S. algae* are unique and rarely documented. This makes rapid treatment and prevention of progression difficult, as physicians often use a trial-and-error method to see which antibiotics improve the patient's condition. Previous publications have reported that *S. algae* are susceptible to aminoglycosides, carbapenems, erythromycin, and fluoroquinolones and has variable susceptibility to penicillin and cephalosporins (with reports of resistance to first and second-generation cephalosporins) [3]. Thus, the best options include ß-lactams, aminoglycosides, and quinolones [8]. In one study, empiric ceftriaxone and doxycycline were started, and then the medication was switched to ciprofloxacin after getting culture and susceptibility results [9]. Despite a low minimum inhibitory concentration to ciprofloxacin, the patient showed minimal improvement and ended up receiving an above-knee joint leg amputation, suggesting that fluoroquinolones are not ideal for the primary treatment of *S. algae*. Another study documented the use of ampicillin and gentamicin during their hospital stay and then discharged the patient with oral ampicillin [8]. This combination resulted in a completely healed wound over the course of two months. This supports the notion that ß-lactam antibiotics are a good treatment option. Another study involved a female patient with an allergy to penicillin. She was started on empiric levofloxacin; however, over the course of 48 hours, her condition deteriorated, so meropenem was used instead. Resolution of the infection was achieved with the use of meropenem (14 days) and linezolid (seven days) [3]. Using broad-spectrum ß-lactam medication (i.e., ceftazidime) halts the progression of the infection.

## Conclusions

This case was reported by the treating physicians and medical students involved because it was the first case of the atypical soft tissue infection caused by *S. algae* that would be reported in Citrus County, Florida. Additionally, this report aims to contribute to the treatment regimen used in our case and potentially offer suggestions for treatment guidelines when dealing with *S. algae* infections, as nothing currently exists. It is crucial we address this lack of standardized treatment and even consider the use of empiric antibiotics in patients who present with soft tissue infections complicated by exposure to any bodies of water in a tropical or warm water environment, as it can delay progression and improve patient outcome in a timelier manner.
